# The association between EGFR variant III, HPV, p16, c-MET, EGFR gene copy number and response to EGFR inhibitors in patients with recurrent or metastatic squamous cell carcinoma of the head and neck

**DOI:** 10.1186/1758-3284-3-11

**Published:** 2011-02-27

**Authors:** Nicole G Chau, Bayardo Perez-Ordonez, Katherine Zhang, Nhu-An Pham, James Ho, Tong Zhang, Olga Ludkovski, Lisa Wang, Eric X Chen, Ming-Sound Tsao, Suzanne Kamel-Reid, Lillian L Siu

**Affiliations:** 1 Division of Medical Oncology and Hematology, Princess Margaret Hospital, University Health Network, Toronto, Ontario, Canada; 2 Department of Pathology, Princess Margaret Hospital, University Health Network, Toronto, Ontario, Canada; 3 Advanced Molecular Profiling Laboratory, Princess Margaret Hospital, University Health Network, Toronto, Ontario, Canada; 4 Department of Biostatistics; Princess Margaret Hospital, University Health Network, Toronto, Ontario, Canada

## Abstract

**Background:**

We examine the potential prognostic and predictive roles of EGFR variant III mutation, EGFR gene copy number (GCN), human papillomavirus (HPV) infection, c-MET and p16^*INK4A *^protein expression in recurrent or metastatic squamous cell carcinoma of the head and neck (R/M SCCHN).

**Methods:**

We analyzed the archival tumor specimens of 53 patients who were treated in 4 phase II trials for R/M SCCHN. Two trials involved the EGFR inhibitor erlotinib, and 2 trials involved non-EGFR targeted agents. EGFRvIII mutation was determined by quantitative RT-PCR, HPV DNA by Linear Array Genotyping, p16 and c-MET protein expression by immunohistochemistry, and EGFR GCN by FISH.

**Results:**

EGFRvIII mutation, detected in 22 patients (42%), was associated with better disease control, but no difference was seen between erlotinib-treated versus non-erlotinib treated patients. EGFRvIII was not associated with TTP or OS. The presence of HPV DNA (38%), p16 immunostaining (32%), c-MET high expression (58%) and EGFR amplification (27%), were not associated with response, TTP or OS.

**Conclusion:**

EGFRvIII mutation, present in about 40% of SCCHN, appears to be an unexpected prognostic biomarker associated with better disease control in R/M SCCHN regardless of treatment with erlotinib. Larger prospective studies are required to validate its significance.

## Background

The epidermal growth factor receptor (EGFR) is over-expressed in up to 90% of squamous cell carcinoma of the head and neck (SCCHN) and has been postulated to be a key molecular target in this malignancy [1]. EGFR signal transduction leads to cell proliferation, invasion, angiogenesis and metastasis [2]. EGFR overexpression and aberrant EGFR gene copy number (EGFR GCN) have been associated with poorer prognosis and disease-specific survival in SCCHN [1,3,4]. Therapies targeted against EGFR have demonstrated modest activity either alone or in combination with chemotherapy in both locally advanced [5] and recurrent and/or metastatic SCCHN [6-10]. No validated biomarkers exist to predict the response to EGFR inhibitors in SCCHN.

The most common EGFR truncation mutation, EGFR variant III (EGFRvIII), harbors an in-frame deletion of exons 2 to 7 (801 bp), resulting in a truncated extracellular EGF-binding domain that is constitutively activated and ineffectively ubiquinated [11,12]. EGFRvIII is found in many human cancers and is present in ~40% of glioblastomas and 5% of lung squamous cell carcinomas, where it confers tumorigenicity and dose-dependent resistance to gefitinib in pre-clinical models [13,14]. The prevalence of EGFRvIII in SCCHN was first reported as 43% in one study of 33 SCCHN tumors [15]. EGFRvIII-transfected SCCHN cells had decreased apoptosis in response to cisplatin and decreased growth inhibition following treatment with the EGFR monoclonal antibody cetuximab compared with controls [15]. EGFRvIII is an interesting therapeutic target because unlike wild-type EGFR, EGFRvIII is not found in normal tissue. EGFRvIII is proposed to account for limitations in response to current EGFR inhibitors, however in patients with SCCHN tumors harboring EGFRvIII response to EGFR tyrosine kinase inhibition (TKI) is unknown.

HPV infection is a risk factor for the development of SCCHN. HPV DNA is found in 20-30% of SCCHN and up to 40-66% of SCCHN of the oropharynx [16,17]. HPV positive oropharyngeal tumors are clinically and molecularly distinct from HPV negative tumors [18,19] and associated with a more favorable prognosis [20]. HPV positive status prospectively predicts survival and response to induction chemotherapy and chemoradiation in stage III or IV oropharynx cancers [21,22] and better response to radiotherapy alone [23]. The combination of low HPV titers and high EGFR expression was associated with worse overall survival in oropharynx cancer [22]. Inactivation of pRb by HPV E7 protein results in overexpression of p16 protein, thus p16 immunostaining has served as a surrogate marker for HPV-associated SCCHN. Patients with tumors lacking both p16 expression and HPV (p16-/HPV-) had the worst disease-specific survival compared to tumors with p16+/HPV+, p16-/HPV+ or p16+/HPV- types [24]. Despite the importance of HPV in the pathogenesis and prognosis of SCCHN in response to chemotherapy and radiation, the role of HPV DNA and response to EGFR inhibitors in SCCHN is unclear.

c-MET, a proto-oncogene tyrosine kinase receptor, is overexpressed in SCCHN, and its ligand, hepatocyte growth factor (HGF), stimulates cell proliferation, motility and invasion [25]. c-MET overexpression has been associated with disease progression in oral squamous cell carcinoma (OSCC) [26]. Elevated serum HGF is associated with resistance to chemoradiation and reduced survival [27]. c-MET amplification and mutations of MET confer an invasive phenotype associated with metastases in SCCHN [28]. Ligand-independent constitutive activation of c-MET via its heterodimerization with EGFR has been identified as a contributing mechanism of acquired resistance to cetuximab in SCCHN [29]. The role of c-MET in response to EGFR TKI in the clinical setting in SCCHN is unknown.

In this study, we examine the prevalence of EGFRvIII, HPV, p16, c-MET and EGFR GCN in patients with R/M SCCHN and explore the potential prognostic and predictive roles of these biomarkers in patients treated with or without EGFR TKI. We hypothesized that EGFRvIII and c-MET would be associated with poorer prognosis or response to EGFR TKI, while HPV and p16 expression would predict improved clinical outcomes and response to treatment.

## Methods

### Patients

We obtained approval from the University Health Network Research Ethics Board to evaluate the archival formalin-fixed paraffin embedded (FFPE) tumor specimens of patients with R/M SCCHN who were treated in four phase II trials for R/M SCCHN at Princess Margaret Hospital conducted from 2000-2005. Two of the four trials involved the EGFR TKI erlotinib (phase II trial of erlotinib [8], phase II trial of erlotinib and cisplatin [7]) and the remaining two trials used other non-EGFR targeted agents (phase II trial of the kinesin spindle protein inhibitor ispinesib [30], phase II trial of the multi-kinase antiangiogenic inhibitor sorafenib [31]). The medical records and case report forms were reviewed to obtain patient demographics, primary tumor site, treatment details and clinical outcome (response rate, time to progression and overall survival).

### Specimen Characteristics

Archival FFPE tumor specimens were available in 35 of 48 patients (73%) treated with erlotinib and 18 of 37 (49%) patients treated with non-EGFR targeted agents. H&E stained sections were examined by a histopathologist (B.P-O.) to confirm the presence of >80% tumor in the specimens evaluated.

### Assay Methods/Molecular Assays

#### EGFRvIII Mutation Detection

##### RNA Isolation

RNA was isolated in tumor area on the FFPE slides guided by H&E-stained serial sections. The tissues were deparaffinized by xylene and ethanol. Total RNA from paraffin-embedded tissues was extracted using RecoveryAll™ Total Nucleic Acid isolation Kit (Ambion Diagnostics, Streetsville, Ontario, Canada).

##### Real-time RT-PCR

Reverse transcription was done using TaqMan Reverse transcription reagent kit (Roche, Branchburg, New Jersey) according to the manufacturer's protocol. Reverse transcription reaction was done in a total volume of 25 mL including RNA template, 1.25 mL random hexamer, reverse transcription buffer, 5.0 mL dNTP, 5.5 mL MgCl, RNase inhibitor, and M-MLV reverse transcriptase.

Real-time PCR was performed in duplicate in 25 mL reaction volumes using Platinum SYBR Green qPCR SuperMix-UDG (Invitrogen, Carlsbad, California) and a 7900HT instrument (Applied Biosystems, Foster City, California). The amplification conditions were: 50°C for 2 min., 95°C for 2 min., 40 cycles of 95°C for 15 sec. and 60°C for 1 min.

##### Data Analysis of real-time PCR

A mixture of at least eight normal FFPE tissue samples was used as a wildtype, normal control. The EGFRvlll cell line (u373flag*EGFRvlll*) was used as a positive control.

The relative expression of *EGFR *exon 4 to *EGFR *exon 9 was determined using the delta delta Ct (ΔΔCt) method. All samples were run in duplicate, and the mean Ct number was used for data analysis. The difference in Ct values (Δthreshold cycle, ΔCt = Exon 9 Ct - Exon 4 Ct) was calculated for each RNA sample. The ΔCt from the normal tissue mixture was then subtracted from the ΔCt of the test sample to generate a ΔΔCt. A negative result occurs when the fold change (exon 9: exon 4 calculated as 2^- ΔΔCt^) is less than a value of 5. This value was arbitrarily chosen to ensure that no false positives were called. A positive result occurs when the fold change is the same as or greater than that of the positive control (7). When the fold change of tested samples falls between that of the normal control and the positive control (i.e. between 5 and 7) the results are considered inconclusive.

#### HPV DNA Detection

The Roche Linear Array HPV Genotyping kit (Roche Molecular Diagnostics, Pleasanton, California) was used to detect 37 low- and high-risk HPV types from FFPE tissues. In brief, FFPE sections were deparaffinized and DNA was extracted using a column based method (QIAamp, Qiagen, Valencia, California). HPV detection was performed using PCR amplification followed by hybridization of the amplified products to oligonucleotide probes and subsequent colorimetric determination. All experiments included an HPV positive control and an HPV negative control.

HPV DNA by in situ hybridization (ISH) using the INFORM HPV III Family 16 probe (Ventana Medical Systems Inc., Tucson, Arizona) which detects genotypes 16, 18, 31, 33, 39, 35, 45, 51, 52, 56, 58 and 66, was performed according to the manufacturer's guidelines using the Ventana Benchmark automated slide staining system. All experiments included an HPV positive control and an HPV negative control. Slides were scored as positive if a punctate or diffuse pattern of signal were observed in the tumor nuclei.

#### P16 and c-MET Detection

Immunohistochemistry (IHC) for p16 and c-MET using the Ventana Benchmark XT auto-immunostainer (Tucson, Arizona) was performed on FFPE sections cut at 4 mm thick. Standardized staining protocols were provided by Ventana for the CINtec p16 Histology kit (MTM Laboratories Inc, Westborough Massachusetts) and c-MET antibody (SP44, rabbit monoclonal, Ventana Medical Systems Inc., Tucson Arizona). Controls were included in each assay, comprising of positive tissue controls and negative controls. All p16 and c-MET IHC slides were reviewed independently by two observers (B.P.O. and N.G.C.) without knowledge of EGFRvIII, HPV status or clinical outcome. p16 staining in SCCHN is generally observed to be dichotomous and scored as absent (weak or no staining) or present (strong and diffuse staining) [32]. c-MET IHC slides were assigned a semi-quantitative score based on the product of an intensity score (0 = no staining or equal to background, 1 = weak, or 2 = strong) and percent of area stained (0 = 0%, 1 = 1-30%, 2 = 31-60%, 3 = >60%). Sections with an inter-observer variation were reassessed by a double-headed light microscope to achieve consensus.

#### EGFR Gene Copy Number

Archival tumor specimens were analyzed for EGFR GCN using fluorescent in situ hybridization (FISH) as previously described [33,34]. One hundred non-overlapping interphase nuclei were scored for EGFR and CEP7 copy number and classified into six categories (University of Colorado Scoring system) by a reviewer blinded to clinical outcome (O.L.) [35].

### Statistical Methods

Descriptive statistics were used to summarize the study cohort and to estimate the parameters of interest. Ninety-five percent confidence intervals were obtained for estimates of the presence of EGFRvIII, HPV, p16, c-MET and EGFR GCN. Exploratory analyses were performed to characterize the relationships between EGFRvIII, HPV, p16, c-MET and EGFR GCN with baseline patient characteristics and outcomes. Only patients with conclusive EGFRvIII results were included in the correlation analyses. The Kaplan-Meier method was used to estimate the overall survival and time to progression. All biomarkers were examined in univariate analysis of overall survival and time to progression using Cox proportional hazards model. Only those which were significant at 0.10 (two-sided) level in the univariate analysis were entered in the multivariate analysis and markers that remained significant at 0.05 (two-sided) level in the multivariate analysis were considered significant prognostic factors. Statistical analyses were performed using the SAS 9.1 software package (SAS Institute, Cary, North Carolina).

## Results

### Patients

The clinical characteristics of the 53 patients in our study are described in Table 1. For the entire cohort, the overall response rate (CR+PR) to study treatment was 4/53 (7.5%), median time to progression (TTP) was 1.8 months (95% CI 1.6-2.7) and median overall survival (OS) was 5.9 months (95% CI 4.5-8.7). Patients in the erlotinib group had a higher median OS of 7.9 months (95% CI 4.7-9.8) compared to patients in the non-erlotinib group with median OS of 4.2 months (95% CI 2.9-7.0) (p = 0.011). The erlotinib group had a higher TTP than the non-erlotinib group 2.7 months (95% CI 1.6-3.5) vs 1.5 months (95% CI 1.3-1.8) (p = 0.0009).

**Table 1:** Clinical characteristics of the entire study cohort (n = 53)

Clinical Characteristic		Number
Median Age (Range)		56 (15-78)

Gender	Female:Male	12:41

ECOG Performance Status	0:1:2	15:34:4

Locoregional Recurrence	Yes:No	45:8

Distant Metastases	Yes:No	19:34

Primary Tumor Site	Oropharynx	20
	Larynx	14
	Oral cavity	10
	Hypopharynx	2
	Neck mass unknown	4
	Paranasal sinus	3

Histologic Grade	Well differentiated	5
	Moderately differentiated	33
	Poorly differentiated	14
	Infiltrating basaloid	1

Prior Therapy	Chemotherapy	21
	Radiation Therapy	51
	Surgery	41

Race	Asian	8
	Black	42
	Caucasian	9
	Other	1

Smoker	Current	33
	Former	3
	Never	15
	Unknown	2

Erlotinib	Yes: No	35:18

Best Response	Partial response	4
	Stable disease	20
	Progressive disease	24
	Inevaluable	5

#### Expression of EGFRvIII mutation by real-time PCR

As the previously reported immunohistochemistry-suitable antibody [15] against EGFRvIII is no longer available, EGFRvIII expression is analysed using the RT-PCR method. The presence of EGFRvIII mutation was detected in 22 patients (42%) (Table 2), negative in 19 patients and inconclusive in 12 patients (Table 3). The median EGFRvIII fold change was 6.8 (0.56 to 576.36) for all patients, 15.0 (4.1 to 576.36) for patients in the EGFRvIII positive group, 1.8 (0.6 to 4.3) for patients in the EGFRvIII negative group, and 6.5 (6.2 to 6.8) for patients in the inconclusive group.

**Table 2:** EGFRvIII mutation positive detected by RT-PCR (n = 22)

Case	Treatment	Primary Site	Specimen Site	EGFRvIII by RT-PCR	EGFRvIII Fold Changes	HPV DNA by Linear Array	P16 IHC	MET score by IHC	EGFR FISH
1	Erlotinib	hypopharynx	Untreated primary	+	11.33	-	-	High	Low polysomy

2	Erlotinib	oral cavity	Untreated primary	+	7.01	-	-	High	Disomy

3	Erlotinib	larynx	Untreated primary	+	61.77	-	-	Low	Low polysomy

4	Erlotinib	larynx	Local recurrence	+	26.64	-	-	Low	High polysomy

5	Erlotinib	neck mass unknown primary	Untreated lymph node	+	60.04	33 +	+	High	Low trisomy

6	Erlotinib	oral cavity	Untreated lymph node	+	218.49	-	-	High	Amplification

7	Erlotinib	oropharynx	Unknown primary	+	15.66	16 +	+	High	Low trisomy

8	Erlotinib	oral cavity	Local recurrence	+	8.94	-	-	High	Low polysomy

9	Erlotinib + Cisplatin	neck mass unknown primary	Untreated lymph node	+	127.84	16+	NE	NE	Low trisomy

10	Erlotinib + Cisplatin	larynx	Node recurrence	+	576.36	16+	+	High	Disomy

11	Erlotinib + Cisplatin	larynx	Untreated primary	+	8.26	-	-	Low	Failed

12	Erlotinib + Cisplatin	larynx	Untreated primary	+	17.38	-	-	High	Low polysomy

13	Erlotinib + Cisplatin	oropharynx	Untreated primary	+	14.28	16+	+	High	Disomy

14	Erlotinib + Cisplatin	oral cavity	Untreated primary	+	69.68	-	NE	NE	Low polysomy

15	Erlotinib + Cisplatin	oral cavity	Untreated primary	+	11.11	53+, 58+, 6+, 52+	-	High	Low polysomy

16	Sorafenib	oropharynx	Untreated primary	+	7.71	16 +	+	Low	Low polysomy

17	Sorafenib	oropharynx	Untreated primary	+	7.93	16 +	+	Low	Low trisomy

18	Ispinesib	neck mass unknown primary	Untreated lymph node	+	4.12	16+, 53+, 51 +	+	High	High polysomy

19	Ispinesib	oropharynx	Untreated primary	+	15.61	16+, 53+, 33+, 51+, 58 +	-	High	High polysomy

20	Ispinesib	larynx	Untreated primary	+	218.26	-	-	High	Disomy

21	Ispinesib	oropharynx	Local recurrence	+	29.25	16+, 53+, 51+	+	Low	Disomy

22	Ispinesib	hypopharynx	Local recurrence	+	9.31	16+, 53+, 58+, 52+	-	High	Low polysomy

Abbreviations: +, positive; -, negative; NE, not evaluable

**Table 3:** EGFRvIII mutation negative detected by RT-PCR (n = 19) and inconclusive cases (n = 12)

Case	Treatment	Primary Site	Specimen Site	EGFRvIII by RT-PCR	EGFRvIII Fold Changes	HPV DNA by Linear Array	P16 IHC	MET score by IHC	EGFR FISH
23	Erlotinib	larynx	Local recurrence	-	1.76	-	-	High	Low polysomy

24	Erlotinib	larynx	Untreated primary	-	0.95	-	-	NE	NE

25	Erlotinib	oropharynx	Untreated primary	Incon.	NE	16 +	-	Low	Failed

26	Erlotinib	larynx	Untreated	Incon.	NE	-	-	NE	NE

27	Erlotinib	oropharynx	Local recurrence	-	2.56	16 +	-	High	Failed

28	Erlotinib	neck mass unknown primary	Local recurrence	-	1.81	-	-	High	Low polysomy

29	Erlotinib	paranasal sinus	Untreated primary	-	0.56	-	-	High	High polysomy

30	Erlotinib	oropharynx	Untreated primary	-	3.16	16 +	+	High	Low trisomy

31	Erlotinib	larynx	Untreated primary	-	3.17	-	-	High	Low polysomy

32	Erlotinib	oropharynx	Local recurrence	NE	NE	-	+	Low	Low polysomy

33	Erlotinib	larynx	Untreated primary	-	3.16	-	-	High	High trisomy

34	Erlotinib + Cisplatin	oropharynx	Untreated primary	Incon.	NE	Incon.	+	Low	NE

35	Erlotinib + Cisplatin	larynx	Untreated primary	-	4.29	-	-	Low	Low polysomy

36	Erlotinib + Cisplatin	oral cavity	Untreated primary	-	0.9	-	-	Low	High polysomy

37	Erlotinib + Cisplatin	paransal sinus	Local recurrence	Incon.	NE	16+, 53+	Incon.	Low	Low polysomy

38	Erlotinib + Cisplatin	larynx	Local recurrence	Incon.	NE	16+, 53+, 33+, 51+	+	High	Disomy

39	Erlotinib + Cisplatin	paranasal sinus	Local recurrence	-	2.59	6+	-	High	NE

40	Erlotinib + Cisplatin	oral cavity	Untreated primary	-	2.01	-	-	High	Disomy

41	Erlotinib + Cisplatin	oropharynx	Untreated primary	Incon.	NE	-	+	High	Low trisomy

42	Erlotinib + Cisplatin	oral cavity	Untreated primary	Incon.	NE	-	-	High	High polysomy

43	Sorafenib	oral cavity	Untreated primary	Incon.	6.8	-	-	Low	Low polysomy

44	Sorafenib	oropharynx	Untreated primary	-	0.99	-	+	High	Amplification/High trisomy

45	Sorafenib	oral cavity	Local recurrence	Incon.	NE	Incon.	-	Low	Low polysomy

46	Sorafenib	oropharynx	Untreated primary	Incon.	6.22	16 +	+	Low	Low trisomy

47	Sorafenib	larynx	Local recurrence	-	2.28	-	+	High	High polysomy

48	Sorafenib	oropharynx	Untreated primary	-	0.64	-	-	High	Low polysomy

49	Sorafenib	oropharynx	Local recurrence	-	1.4	-	-	High	High polysomy

50	Sorafenib	oropharynx	Local recurrence	-	1.3	-	-	High	High polysomy

51	Sorafenib	oropharynx	Local recurrence	-	1.76	-	+	Low	Monosomy/Disomy

52	Ispinesib	oropharynx	Untreated lymph node	Incon.	NE	-	-	High	Low polysomy

53	Ispinesib	oropharynx	Node recurrence	-	4.05	16 +	-	Low	Amplification

Abbreviations: +, positive; -, negative; Incon., inconclusive; NE, not evaluable

Patients with tumors harboring the EGFRvIII mutation had similar clinical characteristics to patients without the EGFRvIII mutation (Table 4).

**Table 4:** Presence of the EGFRvIII mutation is not significantly associated with any clinical characteristics

Clinical Characteristic	EGFRvIII absent (n = 19)	EGFRvIII present (n = 22)	p-value
Male - no., (%)	16 (84%)	18 (82%)	0.839 (Fisher's)

Age - mean, (+/-SD)	53.5 (+/- 11.6)	55.1 (+/- 14.1)	0.685 (t-test)

Oropharynx - no., (%)	8 (42%)	6 (27%)	0.318 (Chi-square)

Larynx - no., (%)	6 (32%)	6 (27%)	0.763 (Chi-square)

Oral Cavity - no., (%)	2 (11%)	5 (23%)	0.271 (Fisher's)

Distant metastasis - no., (%)	4 (21%)	12 (55%)	0.053 (Fisher's)

Locoregional recurrence - no., (%)	18 (95%)	16 (73%)	0.099 (Fisher's)

Well-moderately differentiated - no., (%)	15 (79%)	14 (64%)	0.325 (Fisher's)
	
Poorly differentiated - no., (%)	4 (21%)	8 (36%)	

Prior chemotherapy - no., (%)	9 (47%)	8 (36%)	0.476 (Chi-square)

Prior radiotherapy - no., (%)	17 (89%)	22 (100%)	0.209 (Fisher's)

Prior surgery - no., (%)	17 (89%)	17 (77%)	0.419 (Fisher's)

Caucasian - no., (%)	15 (79%)	18 (82%)	0.562 (Fisher's)

Erlotinib treatment - no., (%)	12 (63%)	15 (68%)	0.735 (Chi-square)
	
No erlotinib treatment - no., (%)	7 (38%)	7 (32%)	

##### EGFRvIII is associated with disease control

**Figure 1:**
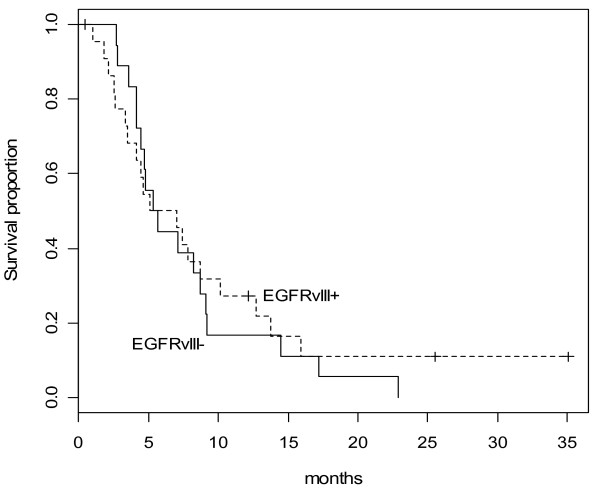
**Overall survival by EGFRvIII mutation status (HR = 0.91, 95% CI: 0.32-2.60, p = 0.85)**.

In univariate analysis, the presence of EGFRvIII was associated with better disease control (Table 5). Median EGFRvIII fold changes were higher for patients with disease control than patients with progressive disease (11.11 vs. 3.16, p = 0.04). No significant difference was observed between erlotinib-treated (p = 0.21) versus non-erlotinib (p = 0.10) treated patients due to the small sample size (Table 5). The presence of EGFRvIII mutation was not associated with TTP (HR 0.94 (95% CI 0.33-2.71), p = 0.91) or OS (HR 0.91 (95% CI 0.32-2.60), p = 0.85) (Figure 1).

**Table 5:** EGFRvIII mutation is associated with disease control

EGFRvIII	Best Response		
	
	Progressive Disease	Disease Control (Partial Response or Stable Disease)	
EGFRvIII absent by ΔΔCt	12 (67%)	6 (33%)	P = 0.0099
	
EGFRvIII present by ΔΔCt	5 (25%)	15 (75%)	(Chi-square)

EGFRvIII mean fold change	10.62	63.76	P = 0.04
	
EGFRvIII median fold change	3.16	11.11	(Wilcoxon)

Erlotinib treated patients			

EGFRvIII absent by ΔΔCt	6 (55%)	5 (45%)	P = 0.21
	
EGFRvIII present by ΔΔCt	3 (33%)	10 (77%)	(Fisher's)

Non-erlotinib treated patients			

EGFRvIII absent by ΔΔCt	6 (86%)	1 (14%)	P = 0.21
	
EGFRvIII present by ΔΔCt	2 (29%)	5 (71%)	(Fisher's)

#### HPV DNA

HPV DNA testing by PCR was positive in 20 patients (38%), negative in 31 patients and inconclusive in 2 patients (Tables 2 and 3). The most prevalent HPV subtype found in our analysis was the high-risk HPV-16 (18/20 cases). The majority of HPV-16 positive tumors were from the oropharynx (12/18). HPV positive tumor status was not significantly associated with disease control (Table 6), TTP (HR 1.19 (95% CI 0.46-3.11), p = 0.722) or OS (HR 0.88 (95% CI 0.34-2.29), p = 0.788).

**Table 6:** HPV by PCR, p16 by IHC, c-MET by IHC are not associated with disease control

	Best Response		
	
	Progressive Disease	Disease Control (Partial Response or Stable Disease)	
HPV PCR negative	14 (50%)	14 (50%)	P = 0.86
	
HPV PCR positive	9 (47%)	10 (53%)	(Chi-square)

P16 IHC negative	14 (47%)	16 (53%)	P = 0.67
	
P16 IHC positive	8 (53%)	7 (47%)	(Chi-square)

c-MET IHC < or = 2	9 (56%)	7 (44%)	P = 0.39
	
c-MET IHC >2	12 (43%)	16 (57%)	(Chi-square)

#### P16

P16 immunoreactivity was detected in 17 patients (32%), absent in 33 patients and inconclusive in 3 patients (Tables 2 and 3). The inter-observer variability rate was 6% and discrepant cases were resolved by consensus review. P16 expression was not associated with disease control (Table 6), TTP (HR 0.50 (95% CI 0.19-1.32), p = 0.16) or OS (HR 0.61 (95%CI 0.24-1.55), p = 0.30).

The discordance between p16 IHC and HPV DNA by PCR was 25% (Table 7). To investigate this further, we performed HPV DNA by ISH. The discordance between p16 IHC and HPV DNA by ISH was lower at 16% (Table 7) and all 7 discordant cases were p16-positive/HPV-ISH-negative. Of these 7 discordant cases, 2 cases were HPV-16 positive by PCR, 4 cases were HPV negative by PCR and 1 case was inconclusive by HPV PCR.

**Table 7:** Concordance of p16 IHC status with HPV by PCR and HPV by ISH

	P16 IHC positive	P16 IHC negative	
**HPV PCR positive**	10	5	P = 0.0016
	
**HPV PCR negative**	5	25	(Chi-Square)

**HPV ISH positive**	6	0	P = 0.0002
	
**HPV ISH negative**	7	31	(Fisher's)

#### C-MET

Forty-nine patients had sufficient tumor samples for evaluation of c-MET. Eighteen patients (63%) had low c-MET scores of 0, 1 or 2 and 31 patients had high c-MET (>2) (Tables 2 and 3). Less than 10% inter-observer variability was observed and discrepant cases were resolved by consensus review. High c-MET was not associated with disease control (Table 6), TTP (HR 1.47 (95%CI 0.56-3.85), p = 0.43) or OS (HR 1.72 (95%CI 0.65-4.56), p = 0.27).

#### EGFR Gene Copy Number

Forty-five patients had sufficient tumor samples for evaluation of EGFR GCN by FISH. High EGFR GCN (amplification and high polysomy) was detected in 13 patients and low EGFR GCN (disomy, low polysomy) was detected in 33 patients (Tables 2 and 3). High EGFR GCN was not predictive for TTP (HR 0.99, p = 0.822) or OS (HR 1.10, p = 0.644). High EGFR GCN was not associated with the presence of EGFRvIII (p = 0.14 Fisher's exact test).

## Discussion

To the best of our knowledge, this is the first study to evaluate the role of EGFRvIII in a cohort of patients with R/M SCCHN treated with or without EGFR TKI. This study confirms that EGFRvIII mutation is common in R/M SCCHN, and may play a role in prognosis. We identified EGFRvIII mutation in 42% of 53 R/M SCCHN tumors. This is in keeping with the first description of EGFRvIII expression by IHC and RT-PCR in 42% of 33 SCCHN tumors sampled [15]. In vitro studies suggest that EGFRvIII mutated SCCHN cell lines are resistant to the anti-EGFR monoclonal antibody cetuximab [15]. In this study, EGFRvIII was not associated with an inferior response to erlotinib therapy. Importantly, we observed a significant association between the presence of EGFRvIII (mean fold change and copy number by RT-PCR) with greater disease control, regardless of treatment with erlotinib, suggesting that perhaps EGFRvIII may have a prognostic role.

The prognostic or predictive significance of the EGFRvIII mutation in response to systemic therapy in patients with SCCHN has not been previously described. The potential prognostic role of EGFRvIII appears to be independent of any clinicopathologic characteristics. This is consistent with another study where EGFRvIII detected by IHC in 234 of 681 locally advanced SCCHN tumors (34%) was associated with increased tumor size but not stage or other clinical factors [36]. In our study, EGFRvIII was not associated with overall survival or TTP. To our knowledge, EGFRvIII has not been linked to survival in SCCHN. EGFRvIII has been described more extensively in glioblastoma where it results in enhanced proliferation and reduced apoptosis effects that are mediated through increased levels of activated Ras [37] and activation of the PI3K pathway [38]. However, the role of EGFRvIII as a prognostic or predictive marker of response to EGFR inhibitors in glioblastoma remains controversial. EGFRvIII and PTEN co-expression was associated with response to EGFR TKI in 26 patients out of a cohort of 49 patients with recurrent glioma and a validation set of 33 patients [39]. EGFRvIII has been reported as a prognostic marker for poorer survival in some studies [40,41], but not in others [42,43]. Conflicting results have been attributed to small sample sizes with incomplete clinical data and varying methods to detect EGFRvIII.

The presence of activating mutations conferring a better prognosis has been reported with EGFR mutations in non-small cell lung cancer (NSCLC) [44] and with PIK3CA mutations in breast cancer [45]. Somatic activating mutations (exon 19 deletion and 21 point mutation) in the EGFR tyrosine kinase domain confer sensitivity to EGFR inhibitors in NSCLC. Patients with these mutations also had improved survival and response to chemotherapy alone [46] or placebo [47]. This suggests that EGFR mutations in NSCLC are a good prognostic factor independent of EGFR TKI, hence it may be more difficult to demonstrate the value of EGFR mutations as predictors of benefit to EGFR TKI [44]. The prognostic value of EGFRvIII in SCCHN needs to be verified, and its role as a predictive marker of response to EGFR inhibitor should remain a relevant therapeutic question.

In this study, the prevalence of HPV, p16 and c-MET expression (38%, 32% and 63% respectively) was in keeping with the literature. We did not observe HPV, p16 and c-MET expression to be predictive of disease control, TTP or OS. This may be due to limitations of a small sample size. Consistent with prior reports [21], HPV-16 was the most common HPV subtype in our study. c-MET is a poor prognostic marker in OSCC [48], however the small proportion (11%) of OSCC in our study precludes any meaningful association.

Limitations of this study include its small sample size, potential bias towards patients with available tumor specimens (larger tumor size), potentially variable fixation and quality of the archival tissues and potential variation in marker status of primary tumor compared with recurrent or metastatic tumors (to our knowledge, this is theoretical and has not been described). Due to the absence of an untreated control group in this study ('control' patients received sorafenib or ispinesib), our results cannot conclusively confirm the prognostic versus predictive value of a biomarker. Although our methods did not use an antibody for EGFRvIII detection, we acknowledge that the use of RT-PCR in FFPE samples has demonstrated superior accuracy relative to IHC tests [49] and may allow greater applicability to settings where frozen tissue is unavailable.

## Conclusion

Predictors of response to EGFR inhibitors in SCCHN remain elusive. Biomarkers are desperately needed to guide patient selection in SCCHN. EGFRvIII remains an interesting tumor-specific target worthy of further exploration as a prognostic or predictive marker of response to EGFR inhibitor therapy in SCCHN. Larger prospective randomized studies are required to distinguish the prognostic and predictive significance of EGFRvIII, HPV, p16, c-MET and EGFR GCN in SCCHN treated with EGFR inhibitors.

## Competing interests

The authors declare that they have no competing interests.

## Authors' contributions

NC participated in the study design, data acquisition, immunohistochemical interpretation and drafted the manuscript. BP-O participated in the histological examination, immunohistochemical interpretation and manuscript preparation. KZ, NAP, JH, TZ, M-ST, SK-R carried out immunostaining, ISH, PCR analysis, and prepared and reviewed the manuscript. OL performed the FISH analysis and reviewed the manuscript. LW performed the statistical analysis. EC participated in patient management and reviewed the manuscript. LS conceived of the study, participated in its design and coordination, and prepared and revised the manuscript. All authors read and approved the final manuscript.
